# May toxicity of amiodarone be prevented by antioxidants? A cell-culture study

**DOI:** 10.1186/1749-8090-7-61

**Published:** 2012-06-28

**Authors:** Ahmet Baris Durukan, Beril Erdem, Elif Durukan, Handan Sevim, Tugce Karaduman, Hasan Alper Gurbuz, Aylin Gurpinar, Cem Yorgancioglu

**Affiliations:** 1 Department of Cardiovascular Surgery, Medicana International Ankara Hospital, Eskisehir Yolu Uzeri, Sogutozu, Ankara, 06520, Turkey; 2 Department Of Biology, Hacettepe University, Faculty Of Science, Beytepe, Ankara, 06800, Turkey; 3 Department Of Public Health, Baskent University Medical Faculty, 79.sokak 7/6 Bahcelievler, Ankara, 06490, Turkey

**Keywords:** Amiodarone, Cytotoxicity, Cell culture, Vitamin C, n-acetyl cysteine

## Abstract

**Background:**

Atrial Fibrillation is the most common arrhythmia encountered following cardiac surgery. The most commonly administered drug used in treatment and prophylaxis is amiodarone which has several toxic effects on major organ functions. There are few clinical data concerning prevention of toxic effects and there is no routinely suggested agent. The aim of this study is to document the cytotoxic effects of amiodarone on cell culture media and compare the cytoprotective effects of commonly used antioxidant agents.

**Methods:**

L929 mouse fibroblast cell line was cultured and 100,000 cells/well-plate were obtained. First group of cells were treated with increasing concentrations of amiodarone (20 to 180 μM) alone. Second and third group of cells were incubated with one-fold equimolar dose of vitamin C and N-acetyl cysteine prior to amiodarone exposure. The viability of cells were measured by MTT assay and the cytoprotective effect of each agent was compared.

**Results:**

The cytotoxicity of amiodarone was significant with concentrations of 100 μM and more. The viabilities of both vitamin C and N-acetyl cysteine treated cells were higher compared to untreated cells.

**Conclusions:**

Vitamin C and N-acetyl cysteine are commonly used in the clinical setting for different purposes in context of their known antioxidant actions. Their role in prevention of amiodarone induced cytotoxicity is not fully documented. The study fully demonstrates the cytoprotective role of both agents in amiodarone induced cytotoxicity on cell culture media; more pronounced with vitamin C in some concentrations. The findings may be projectile for further clinical studies.

## Background

Innumerous studies have been published enclosing treatment and prophylaxis of postoperative atrial fibrillation (AF). Amiodarone is a type 3 anti-arrhythmic agent frequently used for medical cardioversion with varying protocols [1]. It has potential serious side effects in a wide spectrum including photosensitivity, pulmonary toxicity, polyneuropathy, gastrointestinal upset, bradycardia, torsades de pointes (rare), hepatic toxicity, thyroid dysfunction and ocular complications [2-4].

Currently, there are no clinical agents available for prevention of amiodarone induced toxicity. The aim of this study is to underline the toxic effects of amiodarone in cell culture media and propose potential antioxidant agents for clinical use in terms of preventing amiodarone induced toxicity.

## Methods

L929 mouse fibroblastic cell line was plated in 96-well culture plates (Greiner Bio-One, Germany) at an initial density of 100,000 cells/well and incubated in Dulbecco's Modified Eagle Medium/Ham’s F12 (DMEM/F12) (Biochrom AG, Germany) supplemented with 10% fetal bovine serum (FBS) (Biochrom AG, Germany). Following 24 hours of incubation period, the culture medium was removed. The untreated cells were considered as controls. Then, amiodarone hydrochloride (Cordorone®, 150 mg/3 ml, Sanofi-Synthalebo, İstanbul, Turkey) was prepared in nine different concentrations (20 μM, 40 μM, 60 μM, 80 μM, 100 μM, 120 μM, 140 μM, 160 μM, 180 μM). To obtain the studied concentrations, amiodarone was dissolved in the cell culture media. In the first group, the cells were treated with amiodarone alone with the mentioned concentrations. Simultaneously, fresh medium containing one-fold the equimolar dose of amiodarone concentrations of vitamin C (Vitabiol-C®,500 mg/5 ml, İbrahim Etem Ulugay İlaç Sanayi Türk A.Ş, İstanbul Turkey) and N-acetyl cysteine (NAC) (Asist®, 100 mg/ml, Hüsnü Arsan İlaçları A.Ş, İstanbul, Turkey) were prepared. In the second and third groups, in order to study the potential antioxidant effects, cells were preincubated with vitamin-C and NAC for two hours, and then amiodarone was added. After 24 hours of incubation, cell viabilities of the three groups were assessed by the MTT assay and compared with non-pretreated cells. Cell viability was expressed as the relative formazan formation in treated samples as compared to control cells [(A570 treated cells/A570 control cells) x 100%].

### Determination of cell line

L929, a murine aneuploid fibrosarcoma cell line was employed in this study, because it is an established cell line allowing reproducibility of the results, and cells multiply rapidly with an unlimited life span. It is used commonly for multiple purposes including biocompatibility and cytotoxicity tests [5,6].

### Determination of amiodarone concentrations

The concentrations used in cell culture was based on tissue concentration studies. In a survey of 122 patients chronically treated with amiodarone treatment, serum concentrations were measured as 0.4-3.3 (mean 1,7) mg/l, which corresponds to 0.58-4.81 (mean 2.47) μM. Concentrations in myocardial tissue was approximately 30 times the serum concentration (mean 74.1 μM), whereas up to 100 times (247 μM) in various organs [7]. In end stage heart failure, pharmacodynamics differ. Serum concentrations were measured as 0.68 μg/ml which corresponds to 1 μM. Amiodarone concentrations in myocardial tissue was approximately 15 times the serum concentration (15 μM), whereas 150 times (150 μM) in epicardial fat [8]. Similar concentrations used in this study were also used in other cell culture studies made with amiodarone[9].

### MTT assay

MTT assay is a standardized procedure defining viability. This colorimetric method assesses the ability of viable cells to form MTT (3-(4,5-Dimethylthiazol-2-yl)-2,5-diphenyltetrazolium bromide) formazan by the mitochondrial enzyme succinate dehydrogenase. It was originally defined by Carmaichael et al. to assess cytotoxicity [10].

To assess viability, at the end of the designed incubation period, the culture medium was replaced with 200 μl medium containing 0.5 mg/ml MTT and plates were incubated for 3 hours at 37°C. The medium was then removed and replaced with 200 μl of 0.04 M HCl/isopropanol which solubilizes the converted purple dye in culture plates. The absorbance was measured on a spectrophotometer microplate reader (μQuant^TM^, Biotek® Instruments Inc, USA) at a wavelength of 570 nm. Each experiment was repeated 6 times seperately.

### Statistical analysis

Statistical analyses were performed using SPSS software for Windows (version 17.0, Statistical Package for the Social Sciences Inc, Chicago, IL, USA). The viability of cells in different amiodarone doses were compared using repeated measures variance analysis. To compare viability in each concentration according to treatment groups, Kruskal Wallis test was used. In cases of significant difference “bonferoni adjusted Mann–Whitney *u* test” was used to explore the more efficient group.

## Results

When cytotoxicity of amiodarone in different doses was examined, upto 80 μM concentration, decrease in viability was not significant (p < 0.0001). The cytotoxic effects appeared with concentrations of 100 μM and more (p < 0.01). In these concentrations both vitamin C and NAC had cytoprotective effects and lead to increases in viability (p < 0.016) (Table 1). In concentrations of 100, 140 and 180 μM vitamin C and NAC had equal protective effects, whereas in concentrations of 120 and 160 μM, vitamin C was more efficient (p < 0.016).

**Table 1:** The mean cell viabilities (%) after treatment with amiodorane alone and with antioxidant agents

	**Cell viability % (Mean ± SD)**			
Concentration	Amiodarone	Amiodarone + Vitamin C^a^	Amiodarone + NAC^a^	p*
20 μM	117.10 ± 50.75	131.31 ± 36.62	133.86 ± 38.91	0.884
40 μM	130.49 ± 48.70	122.12 ± 24.27	91.83 ± 41.86	0.166
60 μM	97.73 ± 48.08	108.25 ± 16.85	133.65 ± 28.12	0.172
80 μM	81.39 ± 64.45	122.00 ± 19.94	120.34 ± 52.70	0.414
100 μM	59.41 ± 16.43	128.43 ± 30.94	108.74 ± 39.04	0.011
120 μM	26.66 ± 7.40	116.63 ± 23.81	74.93 ± 26.31	0.001
140 μM	19.63 ± 6.81	88.11 ± 29.38	80.33 ± 43.47	0.003
160 μM	16.92 ± 6.46	111.55 ± 24.02	70.18 ± 23.15	0.001
180 μM	14.91 ± 4.32	68.52 ± 25.39	48.58 ± 12.21	0.002

^a^ Vitamin C and NAC were added at one-fold equimolar dose of amiodarone concentrations.

*Kruskal-Wallis test.

NAC: N-acetyl cysteine.

## Discussion

Atrial fibrillation is the most commonly encountered rhythm disorder following cardiac surgery. Amiodarone is a very effective agent used in treatment and prophylaxis of postoperative AF [2,11-13]. The risk of toxicity increases with increasing plasma concentrations. Due to its high liposolubility, it accumulates in adipose tissue and highly perfused organs such as lungs, liver and spleen. This tendency also contributes to its toxic effects besides from cellular mechanisms of toxicity [14].

Free radical formation, direct cytotoxicity, development of lysosomal phospholipidosis and membrane destabilization are the documented possible cellular mechanisms of toxicity, but it is known that the predominant molecular mechanism of amiodarone induced cell death is the trigger caused by oxidative damage [9]. The oxidative stress normally results either due to increased production of reactive oxygen species or decreased antioxidant capacity of cells, of which former is the mechanism in case of amiodarone induced cytotoxicity. Since it is not possible to prevent the development of oxidative stress itself, it would be rational to increase the cellular levels of antioxidants, which is the hypothesis for eveluating the use of antioxidant agents to prevent amiodarone induced cytotoxicity in this study.

To date, there are few data concerning prevention of amiodarone induced cytotoxicity. In some cell culture and experimental animal studies antioxidants, particularly vitamin E was employed and was found to be effective in increasing viability following exposure to toxic doses of amiodarone. Golli-Bennour et al. very clearly demonstrated the cytoprotective role of vitamin E on human cell culture lines [9]. Boldt et. al and Card JW et. al reported attenuation of amiodarone induced pulmonary fibrosis by vitamin E in experimental animal models [15,16]. Agoston et al. studied the antioxidant effect of dietary vitamin E on rat hepatocytes concurrent with amiodarone administration and showed reduction of lysosomal phospholipidosis [17]. Those studies are valuable, since they demonstrate the possible effectiveness of antioxidants for prevention of amiodarone toxicity. But to our knowledge very few studies with vitamin C and NAC were carried out in order to prevent amiodarone induced cytotoxicity.

Vitamin C mediates its antioxidant action possibly via scavenging reactive oxygen and nitrogen species including free radicals such as hydroxyl radicals, superoxide anion, nitrogen dioxide and aqueous radicals. It also acts on nonradical species such as ozone, hypochlorus acid, singlet oxygen, nitroxide, peroxynitrite and nitrosating species. Morover, vitamin C is capable of regenerating small molecule antioxidants such as α-tocopherol, glutathione, urate and β-karotene from their respective radical species [18,19]. At the cellular level, vitamin C prevents the unwanted effects of reactive oxygen species directly by causing an increase in cellular antioxidant enzyme activities and indirectly by reducing oxidized form of vitamin E and glutathione [19]. It is obvious that vitamin-C counteracts on multiple levels of cellular oxidative stress, which we think is the key feature in prevention of amiodarone toxicity.

N-acetyl cysteine has a similar mechanism of antioxidant action which is mediated either directly by scavenging reactive oxygen species or indirectly by production of glutathione [20]. The cysteine component of NAC combines with glutamate and glycine, the precursors in the production of glutathione, in which cysteine is the rate limiting step. Glutathione itself is the most generic antioxidant in the body [21]. Lee et al. demonstrated the preventive role of NAC against contrast induced nephrotoxicity measured by MTT analysis in a similarly designed study on human embryonic kidney cells [20]. We also believe that, NAC prevents amiodarone induced cytotoxicity via glutathione pathway, but molecular analysis should be made for certainity.

When clinical side of the unknown equation of amiodarone induced cytotoxicity is explored, the additive effect of inflammation and oxidative stress which play role in development of arrhythmias should also be considered. Particularly inflammatory response associated with cardiopulmonary bypass is a critical cornerstone following cardiac surgery. Although not fully documented, clinical use of antioxidants may also have favorable effects in this clinical setting. Rodrigo et al. reviewed use of antioxidants for prevention of AF and suggested use of vitamin E and vitamin C hypothesizing that their use not only would minimize the risk of post-cardiac surgery AF, but also increase its treatment success [22]. Carnes et al. reported antioxidant role of vitamin C and documented decreased risk of postoperative AF in patients given prophylactic preoperative and postoperative vitamin C [23]. Similarly, Korantzopaulos et al. studied the effects of vitamin C on recurrence of electrically cardioverted persistent AF and found decreased incidence with vitamin C [24]. In none of the studies, their role in amiodarone treatment was noted. Different from those findings, our study documented the preventive role of vitamin C against amiodarone cytotoxicity in cell culture media.

When NAC is considered in clinical use, Ozaydin et al. reported NAC to have favorable effects in prevention of postoperative AF [25]. They did not use NAC together with amiodarone. Serviddio et al. studied liver toxicity during amiodarone administration in an animal experiment and documented that NAC completely prevented mitochondrial respiratory chain dysfunction and membrane phospholipid damage caused by amiodarone [26]. In cell culture, we also found that NAC prevents amiodarone induced cytotoxicity.

Our findings are valuable in terms of defining cytotoxic effects of amiodarone which occur only in moderate to high doses. In lower doses (80 μM or less), the toxicity is not noted with amiodarone in our study.

The viabilities were measured to be more than 100% in some measurements. There are several explanations for that. The control group is the untreated cells whose spectrophotometric measurements were made after the first 24 hours of incubation period. But for amiodarone alone and amiodarone with antioxidant treated cells, the measurements were made following a second 24 hours of incubation period. The cytotoxic effects of amiodarone began above 100 μM concentration. Moreover, the culture media in which the agents were dissolved, possibly served as a suitable medium for proliferation during the second 24 hours period in lower concentrations.

## Conclusions

The findings of the study demonstrate that vitamin C and N-acetyl cysteine are thought to be effective in terms of decreasing cellular cytotoxicity of amiodarone. The ease and rapidness of using cell culture media for cytoxicity tests are worth noting. Non-clinical, laboratory based studies usually are incapable of answering clinical questions and direct projections are hardly made. But, since the agents used in this study for prevention of cytotoxicity are commonly administered, almost naive agents used for multiple purposes in the routine clinical setting, the findings may be useful for further clinical studies.

### Limitations

L929 is a very frequently used and reliable cell line employed in cytotoxicity tests, so it was the choice of cell line in our study. But human cell lines could also be used for more reliable analysis. Especially, human hepatic and renal cell lines could provide a better understanding of antioxidant behavior of vitamin-C and NAC in terms of prevention of amiodarone induced cytotoxicity, and easier future projections could be made. A similar study with vitamin E was performed by Golli-Bennour on human cell culture lines, and protective effects were demonstrated [9]. But L929 can be considered as a standard cell line employed in cytotoxicity tests, so the results are considered as reliable.

The results could also be supported with further analyses like measurement of lipid peroxidation products which shows the oxidative status of amiodarone treated cells.

## Abbreviations

AF, Atrial fibrillation; NAC, N-acetyl cysteine; MTT, (3-(4,5-Dimethylthiazol-2-yl)-2,5-diphenyltetrazolium bromide).

## Competing interests

The authors declare that they have no competing interests.

## Authors’ contributions

DAB: study concepts, study design, definition of intellectual content, literature research, data acquisition, data analysis, manuscript preparation, manuscript editing. EB: study concepts, study design, definition of intellectual content, literature research, data acquisition, manuscript preparation, manuscript editing. DE: data analysis, statistical analysis, manuscript editing. SH: study concepts, study design, definition of intellectual content, data acquisition, data analysis, manuscript editing. KT: study concepts, study design, definition of intellectual content, data acquisition, data analysis, manuscript editing. GHA: study concepts, study design, definition of intellectual content, literature research, manuscript editing. GA: study concepts, study design, definition of intellectual content, manuscript preparation, manuscript editing, supervision. YC: study concepts, study design, definition of intellectual content manuscript preparation, manuscript editing, supervision. All authors read and approved the final manuscript.

## Authors information

Durukan AB: Specialist in cardiovascular surgery, Making PhD in Biology, particularly dealing with cell culture and stem cells. The standpoint of the study is the look to cell culture from a cardiovascular surgeon’s eye as an easy tool readily applicable for clinical studies.
